# Targeted next generation sequencing identifies two novel mutations in SEPN1 in rigid spine muscular dystrophy 1

**DOI:** 10.18632/oncotarget.13337

**Published:** 2016-11-14

**Authors:** Yi Dai, Shengran Liang, Yan Huang, Lin Chen, Santasree Banerjee

**Affiliations:** ^1^ Department of Neurology, Peking Union Medical College Hospital, Chinese Academy of Medical Sciences, Beijing, Beijing, China; ^2^ BGI-Shenzhen, Shenzhen, Guangdong, China

**Keywords:** SEPN1, Selenoprotein N, SEPN1-RM, compound heterozygotes, respiratory insufficiency, Pathology Section

## Abstract

Rigid spine muscular dystrophy 1 (RSMD1) is a neuromuscular disorder, manifested with poor axial muscle strength, scoliosis and neck weakness, and a variable degree of spinal rigidity with an early ventilatory insufficiency which can lead to death by respiratory failure. Mutations of *SEPN1* gene are associated with autosomal recessive RSMD1. Here, we present a clinical molecular study of a Chinese proband with RSMD1. The proband is a 17 years old male, showing difficulty in feeding, delayed motor response, problem in running with frequent fall down, early onset respiratory insufficiency, general muscle weakness and rigid cervical spine. Muscle biopsy identified increased variability of fiber size with atrophic muscle cells consistent with non-specific myopathic changes. Proband's elder brother presented with same phenotype as the proband and died at the age of 15 years due to acute respiratory failure. Proband's father and mother are phenotypically normal. Targeted exome capture based next generation sequencing and Sanger sequencing identified that the proband was a compound heterozygote with two novel mutations in *SEPN1* gene; a novel missense mutation (c.1384T>C; p.Sec462Arg) and a novel nonsense mutation (c.1525C>T; p.Gln509Ter), inherited from his father and mother respectively. These two mutations are co-segregated with the disease phenotypes in the proband and was absent in normal healthy controls. Our present study expands the mutational spectrum of the *SEPN1* associated RSMD1.

## INTRODUCTION

Rigid spine muscular dystrophy 1 (RSMD1) [MIM# 602771] is an autosomal recessive neuromuscular disease characterized by axial weakness, rigidity of the spine, early and life-threatening respiratory insufficiency [1]. Mutations in *SEPN1* gene are associated with autosomal recessive RSMD1. Patients with SEPN1 related RSMD1 (SEPN1-RM) generally presented with early-onset marked limitation in flexion of the whole dorso-lumbar or cervical spine, followed by contracture of the spinal extensors which finally leading to loss of movement of the spine and the thoracic cage [2]. In addition, it has been reported that patients with SEPN1-RM may also showed limited extension of the elbows and the ankles [3]. Moreover, the muscle histopathology varies among patients with SEPN1-RM, but interestingly they share similar clinical features [4]. The SEPN1-RM is generally manifests within the first 2 years of life and gradually develops respiratory failure in childhood or in early adolescence, necessitating non-invasive ventilation [5]. The progression of proximal muscle weakness and hypotonia is slow and the patients remain ambulant into adulthood [6].

Selenoprotein N (SelN), is encoded by *SEPN1* gene. SelN is an endoplasmic reticulum glycoprotein. SelN is a member of the selenoprotein family, characterized by the presence of a selenium atom in form of a selenocysteine residue [1]. Being an integral integral membrane glycoprotein, SelN localizes to the endoplasmic reticulum [7]. SelN is differentially expressed in skeletal muscle, heart, lung, and placenta [1]. It has been found that SEPN1 protein is expressed at high-level in the diaphragm, which could explain the respiratory impairment that occurs in patients with *SEPN*-related myopathy (*SEPN1*-RM). Previous report also showed that SelN is preferentially expressed in proliferating cells and in fetal tissues in humans and zebrafish [7, 8]. Moreover, the involvement of SelN in the formation and development of muscle muscle is in agreement with the early onset of the disease [7, 9].

In our present study, we analyzed the clinical, histopathological, radiological (MRI), and genetic screening of a Chinese proband affected with autosomal recessive Rigid spine muscular dystrophy 1 (RSMD1). Targeted exome capture based next generation sequencing and Sanger sequencing identified two novel mutations (c.1384T > C; p.Sec462Arg and c.1525C > T; p.Gln509Ter) in *SEPN1* gene in proband which is inherited from father and mother respectively.

## RESULTS

### Clinical investigation

**Figure 1:**
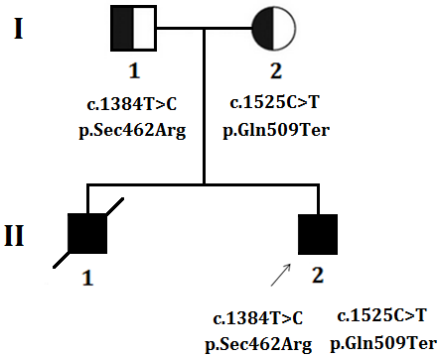
Pedigree The filled symbol indicates the affected individual (Proband), half-filled symbol belongs to carrier of the heterozygous mutation without having disease phenotype (unaffected parents), square represents male and circles female. Arrow indicates the proband.

The proband is the 17 years old boy, the second child of non-consanguineous healthy Chinese parents (Figure 1). After birth, he showed difficulty in feeding with slightly delayed psychomotor development. Since infancy, the proband has been showing the clinical symptoms of neck weakness and dyspnea. In elementary school, he could hardly run and due to muscle weakness he usually fell down frequently during running. In order to get a proper clinical diagnosis with possible treatment, the proband underwent a general and neurological examination, blood test [serum creatine kinase (CK), aspartate aminotransferase (AST), α-hydroxybutyrate dehydrogenase (HBD), lactate dehydrogenase (LDH) levels], electrophysiology, polysomnography and muscle biopsy at Peking union medical college hospital, Beijing, China. Proband's elder brother presented with same clinical manifestations as the proband showed and died at the age of 15 years due to acute respiratory failure.

### Physical examination

Physically, he appeared very thin with myopathic face. He showed slight weakness in his limb girdle muscles (5-/5 of MRC scale) with positive Gowers' sign, spinal rigidity in cervical segment without evident rigidity in the lumber segment.

**Figure 2:**
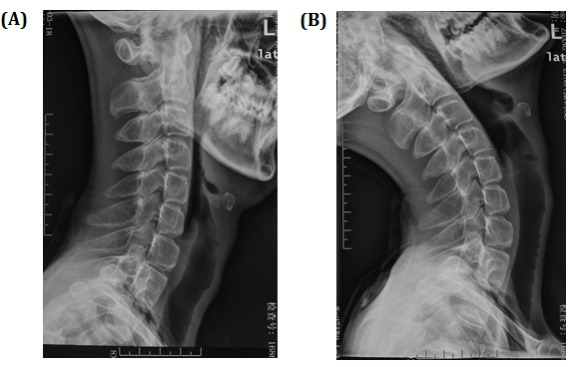
X-ray of cervical flexion and extension

Serum muscle enzymes: CK 110U/L (18-198), AST 28U/L (5-37), HBD 199U/L (72-182), LDH 225U/L (97-270). Myopathic changes has identified by Electromyography. In addition, X-ray of cervical flexion and extension showed that neck flexion is significantly limited (Figure 2).

Diagnostic overnight polysomnography identified that significant elevation in the count of arousal which was 31/night. Hypopnea was 172 times in the night with maximum duration of 142 seconds. Central apnea was 13 times in the night. According to the above mentioned findings, the patient was clinically diagnosed as sleep apnea-hypopnea syndrome with severe hypoxia.

### Pathology of muscle biopsy

**Figure 3:**
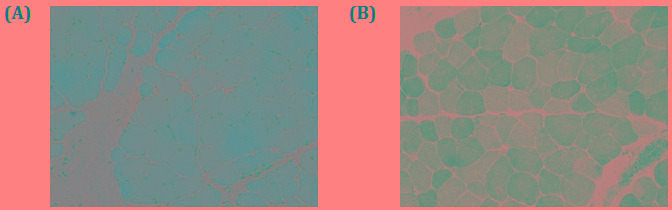
Hematoxylin and eosin (H.E.) staining and NADH staining of muscle.

Muscle biopsy identified increased variability of myofiber size with rounded and atrophied myofibers. Myofibres are devoid of necrosis or regeneration. Additionally, intra-fiber vacuoles or inclusions and inflammatory cell infiltration has not been identified. The type 1 and type 2 myofiber were almost normal in distribution and proportion. In NADH preparation, some myofibers showed slight moth-eaten changes (Figure 3). No other abnormality has been identified by immunohistochemistry staining with dystrophin and emerin. Based on the detailed study by muscle biopsy, the muscle pathology is diagnosed as nonspecific myopathic change.

### Laboratory investigation

#### Mutation analysis

**Figure 4:**
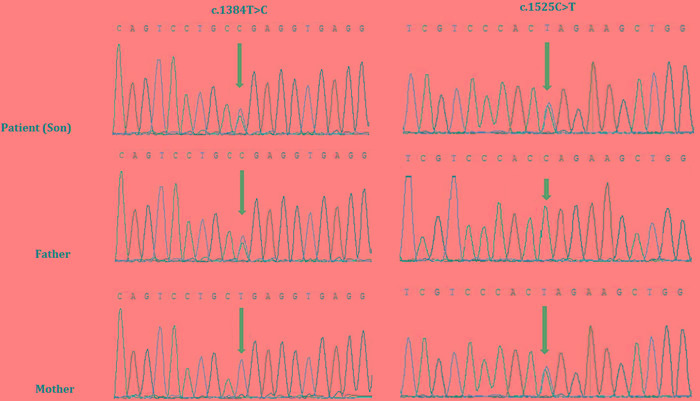
Sanger sequencing of *SEPN1* identified two heterozygous novel mutations, c.1384T > C and c.1525C > T in the proband Proband inherit c.1384T > C from his father and c.1525C > T from his mother. (GenBank Accession: NM_ 020451).

Targeted exome capture based next generation sequencing and Sanger sequencing identified two novel mutations in *SEPN1* gene in the proband. The proband was a compound heterozygote with two novel mutations; a novel missense mutation (c.1384T > C; p.Sec462Arg) and a novel nonsense mutation (c.1525C > T; p.Gln509Ter), inherited from his father and mother respectively (Figure 4). These two mutations are co-segregated with the disease phenotypes in the proband and was absent in 100 healthy controls of same ethnic origin.

## DISCUSSION

In our present study, the proband was compound heterozygous for two novel mutations in *SEPN1* gene, associated with RSMD1. Among these two mutations, a novel missense mutation, c.1384T > C; p.Sec462Arg, proband inherited from his father. However, this missense mutation transformed the selenocysteine codon in exon 10 into an arginine codon (p.U462R). Moreover, p.Sec462 is evolutionarily highly conserved residue among different species. Ana Ferreiro et al., reported that p.U462G of *SEPN1* gene in a homozygous state causes autosomal recessive Multiminicore disease (MmD) with congenital myopathy in a proband of non-consanguineous Portuguese family [7]. Hence, p.Sec462 residue of *SEPN1* gene has a significant role in structure and function of SEPN1 protein. So, subtle changes in the position (p.Sec462) will results into congenital myopathy.

In addition, another novel nonsense mutation, c.1525C > T; p.Gln509Ter, proband inherited from his mother. This nonsense mutation results in the formation of a premature stop-codon, followed by synthesis of a truncated SEPN1 protein. Furthermore, co-segregation study and analysis showed that these two novel mutations were inherited from the heterozygous parents; no *de novo* mutation was identified in the proband. According to the guidelines of American College of Medical Genetics and Genomics (ACMG), these two novel mutations have classified as *“likely pathogenic”* variant [10]. Hence, in this case, these two *“likely pathogenic”* mutations should considered as disease causing mutations in this proband.

The diagnostic clinical symptoms showed by the proband are typical of SEPN-RM. The proband showed early onset axial muscle weakness and gradually develop scoliosis and respiratory insufficiency. In due course of time, the proband presented with significant morphological changes in muscle pathology as variability in myofiber size as well as slight moth-eaten change, compatible with nonspecific myopathic change. In our present study, no minicore, Mallory body-like inclusions or fiber-type disproportion were identified in the proband. However, clinical diagnosis of *SEPN1* associated RSMD by muscle biopsy is very challenging. In addition, detailed or comprehensive analysis of the SelN expression may also be a significant factor for clinical diagnosis of RSMD.

Moreover, it has been reported that RSMD patients with mutations in *SEPN1* are ambulant and present a milder muscular dystrophy without the alteration of the basal membrane and the levels of serum creatine kinase is almost normal [11]. RSMD patients with *SEPN1* mutation are often associated with reduced vital capacity and progressive nocturnal hypoventilation requiring ventilator support, which is correlated with the direct consequence of *SEPN1* dysfunction. Hence, based on the above mentioned reports, the most obvious explanation is that *SEPN1* may play the major significant role in maintaining the redox environment in the cell and preventing it from oxidant damage which in turn maintain the normal physiology of skeletal muscles [11]. In addition, the SelN may also significantly associated with cellular defense against oxidative stress as depletion of SelN results into increase the intracellular oxidant activity and excessive oxidation of proteins [12].

It has been reported that, *SEPN1*-associated RSMD is an autosomal recessive disorder with variable penetrance and sex-linked expression [13]. The high incidence of *SEPN1*-associated RSMD in consanguineous families or in heterozygous parents (carrier) suggesting that homozygous or compound heterozygous mutations in the *SEPN1* gene is causing RSMD which in turn support autosomal recessive inheritance of the disease [8]. Moreover, autosomal dominant inheritance has also been described in patients with SEPN1-related RSMD [13].

Multiminicore disease (MmD) is genetically and clinically heterogeneous congenital disorder of skeletal muscle. The classic MmD is associated with the mutations of SEPN1, characterized by axial muscle weakness which gradually leads to severe, life-threatening respiratory insufficiency and scoliosis, with an autosomal recessive mode of inheritance. Muscle biopsy of patients with classic MmD shows multiple, poorly circumscribed, short areas of sarcomere disorganization and mitochondria depletion (areas termed ‘minicores’) in most muscle fibers [14]. However, dystrophic clinical features, such as muscle fiber necrosis or regeneration or significant endomysial fibrosis, are completely absent in multiminicore disease [15].

Venance et al. [16] reported a patient identified with rigid spine muscular dystrophy having homozygous mutation in the SEPN1 gene. The patient was presented with cor pulmonale characterized by rapidly progressive respiratory and right heart failure with cough, orthopnea, and daytime sleepiness. The patient was also cyanotic with bibasilar crackles, hepatomegaly, pitting edema, severe nocturnal hypoventilation, and prolonged apneic episodes along with associated milder features included restricted neck flexion, thoracolumbar scoliosis, and mild truncal and proximal limb weakness. But patient's two sibs who were heterozygous carriers of the mutation had mild neck restriction [16]. Hence, the interfamilial phenotypic spectrum exists among affected family members with *SEPN1* related RDMD1.

In conclusion, patients with mutations in *SEPN1* are predominantly manifested with axial myopathy, severe scoliosis, and early respiratory failure with almost always include minicore lesions. In summary, in our present study, we illustrates clinical, histopathological, radiological (MRI), and genetic screening of *SEPN1*-related myopathy. It also adds novel mutations to the limited number of fully described pathogenic *SEPN1* variants.

## PATIENTS AND METHODS

### Patient

Informed consent was obtained from the parents of the proband for the publication of clinical and radiological data with cytogenetic and molecular genetic analyses. Patient diagnosis was established through examination by physician, review of medical records and reliable family information. Patient materials were collected with written informed consent using protocols approved by an institutional review board that complies with all principles of the Declaration of Helsinki Principles Accord. Genomic DNA was extracted from peripheral blood by using QIAamp Blood DNA mini Kit (Qiagen, Hilden, Germany) according to the manufacturer's instructions.

### Target exome capture based next generation sequencing

#### Next generation sequencing

DNA sample obtained from the proband was sequenced using Microarray-based next-generation sequencing. A custom Sequence Capture Human Array from Roche NimbleGen (Madison,USA) was designed to capture a 361,992 kb region containing 1,982 exons (including the 100 bp flanking either side) of 68 genes known to be associated with hereditary diseases including nerve and muscles, are enlisted in supplementary Table S1. The 68-gene panel yielded an average of 249514 reads per sample, with approximately 74.89% mapping to the targeted regions. The average mean depth for the targeted regions was 309.09 ± 20.7; 98.69 ± 2.5% of the exons had a coverage ≥30 reads. The procedure for preparation of libraries was consistent with standard operating protocols. In each pooling batch, 10 to 33 samples were sequenced simultaneously on Illumina HiSeq2500 Analyzers (Illumina, San Diego, USA) for 90 cycles. Image analysis, error estimation, and base calling were performed using Illumina Pipeline software (version 1.3.4) to generate raw data. The raw reads were screened to generate - cleanreads‖ followed by established filtering criteria. Clean reads with a length of 90 bp were aligned to the reference of human genome from the NCBI database (Build 37) using the Burrows Wheeler Aligner (BWA) Multi-Vision software package with output files in - bam‖ format. The bam data were used for reads coverage in the target region and sequencing depth computation, SNP and INDEL calling, and CNV detection. First, a novel three-step computational frame work for CNV was applied. Then, SNPs and INDELs were called using SOAPsnp software and Sam tools pileup software, respectively. A SNP or INDEL would be filtered if it could not follow the criterion: supported by at least 10 reads and > 20% of the total reads. The frequency filter was set at 0.05. If a SNP frequency was more than 0.05 in any of the four databases (dbSNP, Hapmap, 1000 Genomes Project, the 124 healthy reference samples sequenced in this study), it would be regarded as a polymorphism instead of a causative mutation. Last, SNVs were retrieved in the The Human Genome Mutation Database and the Leiden Open Variation Database, and then labeled as reported or novel.

### Method of mapping, genotype, SNP calling and indel calling

Image analysis and base calling were performed using the Illumina Pipeline. Indexed primers were used for the data fidelity surveillance. SOAP aligner (soap2.21) was used to align clean reads to the human reference genome (hg19) with maximum 3 mismatches, the parameters were set as ‘-a -b -D -o -u -2 -t -v 3 -l 35 -s 40 -m 0 -x 500 -p 4 -r 1 -n 0’. Based on the results from SOAPaligner, software SOAPsnp (v1.05) was used to assemble the consensus sequence and call genotypes in target regions. The following parameters were set: -Q k -i -d -o -r 0.0005 -e 0.001 -u -L 90 -T -s -2, where ‘-T’ used the targeted and flanking regions. We filter candidate SNPs with the following criterion: snp quality ≥ 20, sequencing depth ≥ 4-fold, estimated copy number ≤ 2 and the distance between two SNPs is larger than 5 insertions and deletions (indels) in the exome regions were identified through the sequencing reads. We aligned the reads to the reference genome by BWA (0.6.2), the parameters were set as ‘-I -L -l 31 -i 15 -k 2 -t 6 -e 63’, and passed the alignment result to GATK, to identify the breakpoints, the parameters were set as ‘mismatch Fraction = 0.05, lod = 5, maxReadsF or Realignment = 30000, maxReadsInRam = 1000000’.

### Direct Sequencing for *SEPN1*

#### Sanger sequencing

To validate true positive of the mutation, Sanger sequencing was performed. Primers flanking the candidate loci (Forward primer 5’-CCGCCCCAGGACAAGTAAAT-3’, Reverse primer 5’-GGAGGAGCTGTTGAAGCCAT-3’) were designed based on the reference genomic sequences of Human Genome from GenBank in NCBI and synthesized by Invitrogen, Shanghai, China. PCR amplification was carried out in ABI 9700 Thermal Cycler. PCR products were directly sequenced on ABI PRISM 3730 automated sequencer (Applied Biosystems, Foster City, CA, USA). Sequence data comparisons and analysis were performed by DNASTAR SeqMan (DNASTAR, Madison, Wisconsin, USA).

## SUPPLEMENTARY MATERIALS TABLE


