# Supplementary material for: Targeted next generation sequencing identifies two novel mutations in SEPN1 in rigid spine muscular dystrophy 1
Source: Oncotarget. 2016 Nov 14;7(51):83843–9. doi: 10.18632/oncotarget.13337 (PMC5356628; doi:10.18632/oncotarget.13337)
Supplement: Supplementary file 1 [file oncotarget-07-83843-s001.docx]

**Supplementary Table:**

Table S1: Gene panel: Name of genes, disorder and mode of inheritance (AD, Autosomal Dominant; AR, Autosomal Recessive; XL, X- chromosome Linked).

| **Disease Name** | **Name of Genes** | **Inheritance**  **Pattern** |
| --- | --- | --- |
| CRYAB-related Myofibrillar Myopathy | *CRYAB* | AR |
| Myofibrillar Myopathy 4 | *LDB3* | AD |
| Myofibrillar Myopathy 6 | *BAG3* | AD |
| Nemaline Myopathy 1 | *TPM3* | AD,AR |
| Nemaline Myopathy 2 | *NEB* | AR |
| Nemaline Myopathy 3 | *ACTA1* | AD |
| Nemaline Myopathy 4 | *TPM2* | AD |
| Nemaline Myopathy 5 | *TNNT1* | AR |
| Nemaline Myopathy 6 | *KBTBD13* | AD |
| Nemaline Myopathy 7 | *CFL2* | AR |
| Salih Myopathy | *TTN* | AR |
| Myopathy with Deficiency of ISCU | *ISCU* | AR |
| X-Linked Centronuclear Myopathy | *MTM1* | XL |
| Hereditary Inclusion Body Myopathy 2 | *GNE* | AR |
| Inclusion Body Myopathy with Paget Disease of Bone&Frontotemporal Dementia | *VCP* | AD |
| Myotonia Congenita | *CLCN1* | AD,AR |
| Paramyotonia Congenita | *SCN4A* | AD |
| Central Core Disease | *RYR1* | AD,AR |
| Multiminicore Disease | *SEPN1* | AR |
| Miyoshi Distal Myopathy (Miyoshi Myopathy) | *DYSF* | AR |
| Myosin Storage Myopathy | *MYH7* | AD |
| Collagen VI-deficient Congenital Muscular Dystrophy | *COL6A1, COL6A2, COL6A3* | AD,AR |
| Brody Myopathy | *ATP2A1* | AR |
| Distal Myopathy 2 | *MATR3* | AD |
| Distal Myopathy 4 | *FLNC* | AD |
| Hypokalemic Periodic Paralysis | *CACNA1S* | AD |
| LAMA2-Related Congenital Muscular Dystrophy | *LAMA2* | AR |
| SYNE1-related Congenital Muscular Dystrophy | *SYNE1* | AR |
| Becker Muscular Dystrophy | *DMD* | XL |
| Muscular dystrophy-dystroglycanopathy type B1 | *POMT1* | AR |
| Muscular dystrophy-dystroglycanopathy type B2 | *POMT2* | AR |
| Muscular dystrophy-dystroglycanopathy type B4 | *FKTN* | AR |
| Muscular dystrophy-dystroglycanopathy type B5 | *FKRP* | AR |
| Muscular dystrophy-dystroglycanopathy type B6 | *LARGE* | AR |
| Muscular dystrophy-dystroglycanopathy type B3 | *POMGNT1* | AR |
| Limb-Girdle Muscular Dystrophy type 2C | *SGCG* | AR |
| Limb-Girdle Muscular Dystrophy type 2D | *SGCA* | AR |
| Limb-Girdle Muscular Dystrophy type 2E | *SGCB* | AR |
| Limb-Girdle Muscular Dystrophy type 2F | *SGCD* | AR |
| Limb-Girdle Muscular Dystrophy type 2A | *CAPN3* | AR |
| Limb-Girdle Muscular Dystrophy type 2G | *TCAP* | AR |
| Limb-Girdle Muscular Dystrophy type 2H | *TRIM32* | AR |
| Limb-Girdle Muscular Dystrophy type 2L | *ANO5* | AR |
| Limb-Girdle Muscular Dystrophy type 2Q | *PLEC* | AR |
| Limb-Girdle Muscular Dystrophy type 2R | *DES* | AR |
| Limb-Girdle Muscular Dystrophy type 1A | *MYOT* | AD |
| Limb-Girdle Muscular Dystrophy type 1B | *LMNA* | AD |
| Limb-Girdle Muscular Dystrophy type 1C | *CAV3* | AD |
| Limb-Girdle Muscular Dystrophy type 1E | *DNAJB6* | AD |
| X-linked Emery-Dreifuss Muscular Dystrophy 1 | *EMD* | XL |
| X-linked Emery-Dreifuss Muscular Dystrophy 6 | *FHL1* | XL |
| Emery-Dreifuss muscular dystrophy 4 | *SYNE1* | AD |
| Emery-Dreifuss muscular dystrophy 5 | *SYNE2* | AD |
| Emery-Dreifuss muscular dystrophy 7 | *TMEM43* | AD |
| Congenital myasthenic syndrome with tubular aggregates 2 | *DPAGT1* | AD |
| AGRN-Related Congenital Myasthenic Syndrome | *AGRN* | AR |
| CHAT-Related Congenital Myasthenic Syndrome | *CHAT* | AR |
| CHRNA1-Related Congenital Myasthenic Syndrome | *CHRNA1* | AR |
| CHRNB1-Related Congenital Myasthenic Syndrome | *CHRNB1* | AR |
| CHRND-Related Congenital Myasthenic Syndrome | *CHRND* | AR |
| CHRNE-Related Congenital Myasthenic Syndrome | *CHRNE* | AR |
| COLQ-Related Congenital Myasthenic Syndrome | *COLQ* | AR |
| DOK7-Related Congenital Myasthenic Syndrome | *DOK7* | AR |
| GFPT1-Related Congenital Myasthenic Syndrome | *GFPT1* | AR |
| MUSK-Related Congenital Myasthenic Syndrome | *MUSK* | AR |
| RAPSN-Related Congenital Myasthenic Syndrome | *RAPSN* | AR |
